# Research on Computation Offloading and Resource Allocation Strategy Based on MADDPG for Integrated Space–Air–Marine Network

**DOI:** 10.3390/e27080803

**Published:** 2025-07-28

**Authors:** Haixiang Gao

**Affiliations:** College of Information Engineering, Shanghai Maritime University, Shanghai 200135, China; gaohaixiang@stu.shmtu.edu.cn

**Keywords:** computation offloading, resource allocation, MADDPG, integrated space–air–marine network, mobile edge computing

## Abstract

This paper investigates the problem of computation offloading and resource allocation in an integrated space–air–sea network based on unmanned aerial vehicle (UAV) and low Earth orbit (LEO) satellites supporting Maritime Internet of Things (M-IoT) devices. Considering the complex, dynamic environment comprising M-IoT devices, UAVs and LEO satellites, traditional optimization methods encounter significant limitations due to non-convexity and the combinatorial explosion in possible solutions. A multi-agent deep deterministic policy gradient (MADDPG)-based optimization algorithm is proposed to address these challenges. This algorithm is designed to minimize the total system costs, balancing energy consumption and latency through partial task offloading within a cloud–edge-device collaborative mobile edge computing (MEC) system. A comprehensive system model is proposed, with the problem formulated as a partially observable Markov decision process (POMDP) that integrates association control, power control, computing resource allocation, and task distribution. Each M-IoT device and UAV acts as an intelligent agent, collaboratively learning the optimal offloading strategies through a centralized training and decentralized execution framework inherent in the MADDPG. The numerical simulations validate the effectiveness of the proposed MADDPG-based approach, which demonstrates rapid convergence and significantly outperforms baseline methods, and indicate that the proposed MADDPG-based algorithm reduces the total system cost by 15–60% specifically.

## 1. Introduction

With the rapid advancement and extensive deployment of Internet of Things (IoT) technologies, the Marine Internet of Things (M-IoT) has become a critical enabler of a broad range of maritime applications, including oceanographic monitoring, marine resource exploration, environmental surveillance and intelligent maritime traffic management [1,2,3]. By interconnecting diverse marine sensors, unmanned platforms, and communication terminals, the M-IoT facilitates enhanced situational awareness and supports intelligent decision-making in complex ocean environments [4,5,6,7].

However, the inherently harsh and heterogeneous nature of marine settings poses significant challenges to the computational efficiency and operational sustainability of M-IoT systems. These challenges are exacerbated by the widespread reliance on resource-constrained edge devices, intermittent and high-latency satellite communication links, and limited onboard energy supplies [8]. Collectively, these constraints impede real-time, efficient task execution, diminish system responsiveness, and restrict the scalability and adaptability of distributed computing architectures in maritime contexts.

Meanwhile, in real-world deployment, the strict payload and energy restrictions of UAV platforms impose significant limits on onboard neural inference and edge server integration, as continuous policy execution and GPU-based acceleration can substantially reduce flight endurance. Maritime communication links are subject to intermittent obstruction, long propagation delays and fluctuating bandwidth, requiring robust channel estimation, adaptive modulation and coding schemes and predefined fallback policies during connectivity outages. Moreover, unanticipated environmental variations demand real-time policy adaptation through lightweight online fine-tuning or federated meta-reinforcement learning, implemented with careful management of computational and communication budgets. Finally, when extended to multi-UAV operations, decentralized or hierarchical training frameworks with periodic aggregation of local experiences can preserve the collective performance without overwhelming the limited high-capacity links available at sea.

## 2. Related Work

Resource management and computation offloading optimization problems usually involve non-convex optimization with multi-objective coupling, such as power control, user association and arithmetic allocation. To address this problem, several classes of traditional algorithms are applied by researchers. For instance, the decomposition optimization algorithm is used to split the large-scale non-convex programming problem into easily solvable subproblems. In [9], the authors consider a binary offloading policy in a wireless-powered multi-user MEC system and propose a joint ADMM-based decomposition algorithm to tackle the combinatorial coupling between offloading decisions and time allocation. In order to overcome the doubly near-far effect, Hu et al. [10] address the “doubly near-far” problem in two-device WPT-powered MEC via a two-phase Lagrangian and bisection scheme that yields closed-form offloading and timing rules, thus minimizing the AP energy under latency constraints. Hassan et al. [11] formulate an MINLP for energy-efficient coverage in 6 G space–air–sea networks and apply Benders’ decomposition, coupled with Dinkelbach’s fractional-programming and ADMM, to achieve near-optimal energy efficiency. Similarly, in [12], the authors study joint task computation and time allocation for blue data in 6 G space–air–sea non-terrestrial networks, formulating a mixed-integer linear program (MILP) for weighted sum-rate maximization across high- and low-antenna gain maritime users. They apply Benders’ decomposition to separate binary offloading decisions from continuous time allocation and use primal decomposition to handle coupling constraints, achieving near-optimal performance with polynomial time complexity. To address the multi-party conflict of interest and pricing problem, many researchers adopt the Stackelberg game and two-way auction model. Hosseini et al. [13] study radio resource allocation in UAV-supported vehicular networks employing NOMA, formulating an MINLP to maximize I2V users’ data rates while minimizing V2V interference under SIC constraints. They propose two low-complexity near-optimal methods: first, a DC (difference-of-concave) approximation-based iterative algorithm for joint power and RB allocation; and second, a Stackelberg game framework in which leaders (I2V users) and followers (V2V users) optimize the transmit power via closed-form KKT solutions, yielding fast convergence and scalability. In [14], the authors focus on joint computation offloading and service pricing in vehicular edge computing (VEC), modeling the interactions among the requesting vehicle, edge server, and cloud as a Stackelberg game, and transform the game into a convex optimization via backward induction, prove the existence and uniqueness of the Nash equilibrium for offloading decisions, and propose a genetic algorithm-based search to derive optimal pricing strategies for edge and cloud servers, demonstrating improved delay–cost trade-offs. Dai et al. [15] study an incentive-oriented two-tier task offloading scheme in marine edge computing networks, introducing a hybrid game-theoretic approach spanning an underwater acoustic tier and an RF tier. They formulate the underwater offloading between the UWS, UUV, and SN as a Stackelberg game and the aerial offloading between the SN and UAV as a double auction, deriving equilibrium offloading and pricing/bidding strategies that maximize each party’s utility. In order to realize real-time adaptive scheduling in the face of time-varying networks with unknown cost functions, Liu et al. [16] study the long-term task completion delay minimization problem in satellite-enabled space–air–ground integrated MEC networks for 6 G. They formulate a stochastic optimization problem and apply Lyapunov optimization to decompose it into per-satellite deterministic subproblems, then they adapt a delayed online learning technique to predict the dynamic task arrivals and queue lengths for use in the cooperative offloading policy, achieving a reduced average delay while maintaining computation efficiency. Xu [17] study fog computation scheduling for marine data feedback under severely limited maritime communication resources, proposing an online gradient-free scheduling algorithm that compresses data at the fog nodes before transmission, and the proposed scheme requires neither explicit cost nor constraint functions, using first-order and second-order estimation to allocate computation resources and thereby improving the communication efficiency while guaranteeing QoS for diverse applications. Meanwhile, the matching algorithm is applied to the computation offloading problem. In [18], Xiao et al. study voyage-based computation offloading in secure maritime edge networks, where both user devices and edge nodes (onships) move along fixed routes. They propose a two-timescale mechanism. On a large timescale, a Hopcroft–Karp-based algorithm precomputes the optimal offloading matches from voyage information, then on a small timescale, real-time task requests use these precalculated matches or local execution based on the resource demand. Simulations with real ship trajectories show marked improvements in the task delay, energy consumption, and traffic cost. Yang et al. [19] study computation task offloading and server selection for vessel terminals in maritime mobile edge computing networks, aiming to minimize both energy consumption and execution delay. They model offloading as a two-step decision and propose a multivessel computation offloading algorithm based on an improved Hungarian matching algorithm, demonstrating significant delay and energy savings via simulation. Although traditional methods are widely used in optimization problems for different communication application scenarios, they often rely on structured assumptions or static models of the problem, showing limitations when facing multi-intelligent agent interactions, partially observable and high-dimension continuous action spaces.

Recently, the application of deep reinforcement learning (DRL) to resource management optimization problems has attracted the attention of researchers. Compared with traditional algorithms, DRL enables direct access to optimal policies in high-dimensional, non-convex, dynamic, and partially observable environments through end-to-end learning without explicit modeling or step-by-step solving. For instance, Nasir and Guo [20] study the dynamic power allocation problem in wireless networks, where each transmitter must choose its transmission power in real time to maximize a weighted sum-rate utility under delayed and incomplete CSI. They introduce a distributed deep Q-learning scheme where each agent collects local CSI and QoS information from neighbors and learns a power control policy robust to feedback delays and channel variations. In [21], Meng et al. address the downlink sum-rate maximization problem in a multi-user cellular network, formulating it as a near-static optimization under maximum power constraints. They propose a multi-agent framework combining three different DRL algorithms to learn distributed power control policies that outperform model-based benchmarks in both the sum-rate and robustness. Alam et al. [22] tackle the non-convex power allocation problem in 6 G interference channels with per-link outage constraints. They first reformulate the problem as a geometric programming instance, then develop a dual deep Q-network approach to stabilize learning, which achieves near-optimal sum-rate performance with much lower computational cost than classical methods. In [23], the authors investigate joint aerial–base-station (AeBS) deployment and computation offloading in a 6 G aerial edge computing network. They model the problem as minimizing the task-processing delay and energy consumption via both deployment control and offloading decisions, and they design a federated deep reinforcement learning (FedDRL) scheme allowing for fast offline training and quick online adaptation to environmental changes. Ye et al. [24] propose a decentralized deep reinforcement learning mechanism for vehicle-to-vehicle (V2V) communications, supporting both unicast and broadcast modes, with carefully designed state, action, and reward functions, achieving scalable, low-overhead resource allocation. In [25], Qi et al. study the service offloading decision for the Internet of Vehicles (IoV), formulating multi-task offloading with data dependencies as a long-term planning problem, and introduce a knowledge-driven deep reinforcement learning framework, supporting offline pre-training at edge servers and continual online federated learning to directly learn optimal offloading policies that adapt to environmental changes. Zhou et al. [26] address the NP-hard two-stage scheduling problem in end–edge–cloud Internet of Everything (IoE) systems by combining Johnson’s rule-based presorting of two-stage tasks with a deep reinforcement learning-enhanced scheduler (DRL-TSS), achieving near-optimal makespan minimization across heterogeneous edge executors. In [27], the authors investigate the content distribution in layered fog radio access networks (FRANs), formulating a minimal delay resource allocation model with in-network caching and cloud–edge cooperation, and propose a cross-layer deep reinforcement learning policy to jointly optimize caching and routing decisions for low-latency content delivery. Wang et al. [28] study adaptive resource allocation in dynamic MEC environments, leveraging an SDN-enabled architecture and a DRL-based resource allocation (DRLRA) scheme to jointly allocate network routes and edge computing resources, thereby minimizing the service time and balancing the load under varying request patterns. The authors of [29] examine the beam allocation in maritime communication systems by discretizing the coverage area into a grid and modeling the beam assignment under unknown CSI as a sequential decision process, and they introduce a virtual queue-based DQN (VQDRL) scheme that adaptively allocates beams to maximize the maritime user data rates. Feng et al. [30] study cooperative computation offloading and resource allocation in blockchain-enabled MEC systems, introducing an asynchronous advantage actor–critic (A3C) deep reinforcement learning algorithm that maximizes both the MEC computation rate and the blockchain transaction throughput by jointly optimizing the offloading decisions, power allocation, block size, and block interval to dynamically learn the optimal policy under channel and queue dynamics. In [31], the authors study joint communication and computation resource management in a space–air–ground–sea integrated maritime network and develop a DQN-based solution to jointly optimize task offloading and resource assignment under dynamic maritime conditions, markedly improving latency and resource utilization compared to heuristic baselines. Resource management and computation offloading optimization problems usually involve non-convex optimization with multi-objective coupling, such as power control, user association and arithmetic allocation. To address this problem, several classes of traditional algorithms are applied by researchers. For instance, the decomposition optimization algorithm is used to split the large-scale non-convex programming problem into easily solvable subproblems. In [32], a two-stage edge server placement algorithm is proposed to optimize safety, efficiency, and adaptability in generative AI-enabled Internet of Vehicles systems, which achieves better load balancing, lower energy use, and reduced latency.

In this paper, computation offloading and resource allocation in an integrated space–air–marine network composed of multiple UAVs equipped with edge servers, an LEO satellite, and heterogeneous maritime IoT devices are investigated. A cloud–edge-device collaborative MEC system supporting partial task offloading under joint latency and energy consumption constraints is first modeled, with the joint association control, power allocation, task partitioning, and resource distribution is formulated as a non-convex mixed-integer optimization problem. To efficiently solve this problem in a dynamic, partially observable environment, it is reformulated as a POMDP and a multi-agent deep deterministic policy gradient (MADDPG)-based algorithm is developed that leverages centralized training with decentralized execution, enabling each agent to learn continuous control policies for offloading decisions and resource allocation. The main contributions of this paper are as follow:

1. A hierarchical cloud–edge-device collaborative MEC system is established, which integrates LEO satellites, UAVs and maritime IoT devices, then a joint optimization problem for task offloading and resource allocation is formulated under latency and energy constraints based on the system model.
2. The optimization problem is reformulated as a partially observable Markov decision process (POMDP) and a multi-agent reinforcement learning framework based on the MADDPG is presented, which enables decentralized agents to perform collaborative offloading decisions through centralized training, effectively handling the non-station dynamics of space–air–marine networks.
3. Extensive numerical simulations are carried out to validate the effectiveness of the proposed MADDPG-based offloading and allocation scheme. The results demonstrate that the algorithm consistently outperforms baseline strategies in terms of the energy-delay tradeoff, convergence rate and overall system efficiency.

The reminder of this paper is structured as follows. The details of the system model and problem formulation are introduced in Section 3. In Section 4, the proposed MADDPG-based algorithm for computation offloading and resource allocation in the integrated space–air–marine network is presented in detail. The performance of the proposed algorithm will be evaluated through simulation experiments and compared with baseline methods in Section 5. Finally, this paper is concluded in Section 6.

## 3. System Model and Problem Formulation

### 3.1. Network Model

**Figure 1:**
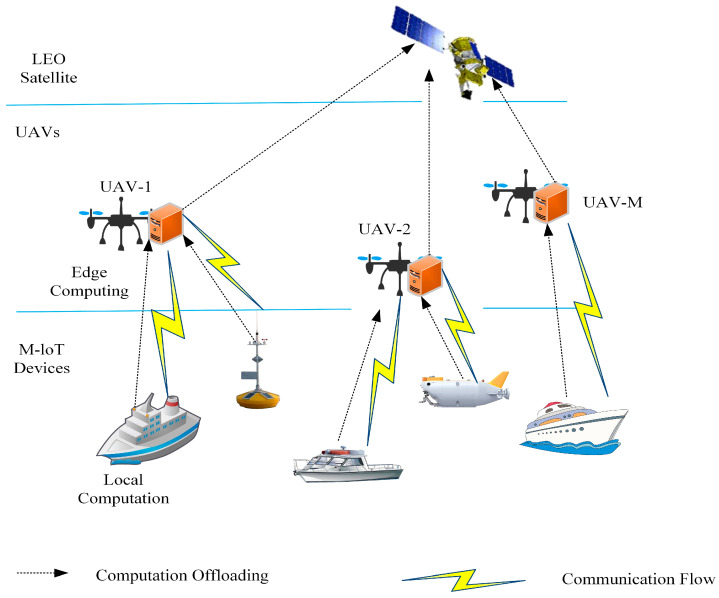
System model diagram.

This section considers a multi-UAV-assisted edge computing system for M-IoT devices, which consists of an LEO satellite, multiple UAVs, and multiple M-IoT devices, including buoys, ships, and various sensors, as shown in Figure 1. Each UAV is equipped with an edge computing server to provide task offloading services for M-IoT devices, and both the UAVs and the M-IoT devices are powered by their own energy reserves. It is assumed that the total M-IoT devices are evenly distributed in the designated sea area, and all the M-IoT devices are within the coverage of the UAVs and the LEO satellite. There are UAVs in the specified sea area, and the number of M-IoT devices and UAVs can be represented by sets N={1,2,⋯,N} and M={1,2,⋯,M}. The entire continuous time is divided into equal time slots. 

### 3.2. Communication Model

In this system, communication between M-IoT devices and UAVs uses FDMA (frequency division multiple access). In a three-dimensional Cartesian coordinate system, the position of M-IoT device *i* can be expressed as $p_{i}(t)=\left[x_{i}(t),y_{i}(t),0\right]$, and UAVs are always flying at a fixed altitude *H*. The position of the *j*-th UAV in time slot *t* can be expressed as $q_{j}(t)=\left[x_{j}(t),y_{j}(t),H\right]$. Therefore, the distance between the *i*-th M-IoT device and the *j*-th UAV is:

(1) $$d_{ij}=\sqrt{H^{2}+{\left[x_{i}(t)-x_{j}(t)\right]}^{2}+{\left[y_{i}(t)-y_{j}(t)\right]}^{2}}\;=\sqrt{{\left\|p_{i}(t)-q_{j}(t)\right\|}^{2}}$$

Assuming the height of the UAV is sufficient for line-of-sight transmission, a typical composite channel containing large and small fading can be employed. Therefore, at each time slot *t*, the channel gain between the *i*-th M-IoT device and the *j*-th UAV can be expressed as:

(2) $$h_{ij}=g_{ij}{\hat{h}}_{ij}$$

$g_{ij}$ and ${\hat{h}}_{ij}$ represent the path loss and Rice fading during information transmission, respectively. In particular, the path loss can be expressed as $g_{ij}=h_{0}d_{ij}^{-\alpha }$, where the $h_{0}$ reference channel gain at the distance $d_{0}=1$ is represented, *α* is the path loss index, which indicates the rate at which the signal attenuates with distance, and the specific value is α=2. Meanwhile, the Rice fading can be given by the following formula.

(3) $${\hat{h}}_{ij}=\sqrt{\frac{M}{M+1}{\bar{h}}_{ij}+\frac{1}{M+1}{\tilde{h}}_{ij}}$$

Here, *M* is the Rice fading factor, which indicates the power ratio between the line-of-sight (LoS) component and the non-line-of-sight (NLoS) component, where a higher M value indicates that the LoS component dominates, resulting in smaller channel fading, ${\bar{h}}_{ij}$ represents the LoS component satisfying $\left|{\bar{h}}_{ij}\right|=1$, and ${\tilde{h}}_{ij}$ represents the NLoS component following the ${\tilde{h}}_{ij}\sim CN(0,1)$ distribution.

To avoid serious co-channel interference, the M-IoT device offloads its computing tasks to the UAV in FDMA mode. If the *i*-th M-IoT device decides to offload its tasks to the *j*-th UAV, the offloading rate of the *i*-th M-IoT device to the *j*-th UAV can be expressed as:

(4) $$R_{ij}=B_{ij}\left(1+\frac{P_{ij}^{u}|{\hat{h}}_{ij}{|}^{2}g_{ij}}{N_{0}}\right)$$

Here, $B_{ij}$ represents the bandwidth allocated to the M-IoT device for the unloading task, $P_{ij}^{u}$ represents the transmission power from the *i*-th M-IoT device to the *j*-th UAV, and $N_{0}$ is the noise power.

### 3.3. Calculation Model

Each M-IoT device has a computation-intensive task. For M-IoT device *i*, the task information can be represented as {*D_i_*, *C_i_*, *T_i_*}, where *D_i_* denotes the input data size of the task for device *i*, *C_i_* represents the number of CPU cycles required to process 1 bit of data, and *T_i_* indicates the maximum delay tolerated for task processing. Considering that each M-IoT device has a large computing task that must be completed within the maximum tolerable delay, the following offloading scheme can be adopted. Due to the limited computational capacity of M-IoT devices, part of the task is processed locally, while another part needs to be offloaded to the UAV nodes for auxiliary computation. If the UAV nodes still cannot fully process the M-IoT device’s task, further offloading to the cloud servers equipped on the LEO satellites is considered.

Specifically, the total task size of M-IoT device *i* can be divided into three parts, represented as $D_{i}^{l},D_{i}^{u}$ and $D_{i}^{s}$, where $D_{i}^{l}$ represents the portion of the task processed on the M-IoT device, $D_{i}^{u}$ denotes the portion processed on the UAV, and $D_{i}^{s}$ represents the portion processed on the LEO satellite. Similar to [9], it is assumed that task partitioning does not introduce additional computational input data, i.e.,

(5) $$D_{i}^{l}+D_{i}^{u}+D_{i}^{s}=D_{i}$$

For example, when $D_{i}^{l}=D_{i}^{s}=0$, the entire task will be offloaded to the UAV, and so on. In this system, three methods can be used to process tasks, including local execution, offloading to the UAV, and offloading to the LEO satellite.

#### 3.3.1. Local Computing Model

The delay for executing the bit-level task $D_{i}^{l}$ is given by the following formula.

(6) $$T_{i}^{l}=\frac{D_{i}^{l}C_{i}}{f_{i}^{l}}$$

Here, $f_{i}^{l}$ represents the CPU clock frequency of the *i*-th M-IoT device.

Accordingly, the energy consumption for executing the computing task locally is:

(7) $$E_{i}^{l}=\kappa_{i}{\left(f_{i}^{l}\right)}^{2}D_{i}^{l}C_{i}$$

Here, $\kappa_{i}$ represents the energy consumption coefficient related to the CPU architecture of the *i*-th M-IoT device, which indicates the energy consumption coefficient per cycle of the CPU of device *i* and depends on the effective switching capacitance of the chip architecture [10].

#### 3.3.2. Edge Computing Model

If an M-IoT device offloads part of its computational tasks to a UAV, the resulting delay consists of three parts: the communication time to transmit the task to the UAV, the delay in executing the task on the UAV, and the time required to transmit the computation results back to the M-IoT device. The computational cost is determined by the energy consumption and task execution delay of the M-IoT device. Due to the relatively small size of the data being returned, the delay and energy consumption in terms of transmitting the results are generally negligible [30].

Let *a_ij_* represent the connectivity indicator variable between the *i*-th M-IoT device and the *j*-th UAV, indicating whether it satisfies the following:

(8) $$a_{ij}\in \{0,1\},\forall i\in N,j\in M$$

Here, *a_ij_* = 1 indicates that the *i*-th M-IoT device decides to offload its task to the *j*-th UAV; otherwise, *a_ij_* = 0.

It is worth noting that each M-IoT device can connect to at most one UAV, leading to the following constraint:

(9) $$\sum_{j=1}^{M}a_{ij}⩽1$$

For the *i*-th M-IoT device, the processing delay on the UAV consists of the time for task offloading transmission and the task processing time. Therefore, it can be calculated as:

(10) $$T_{i}^{u}=\sum_{j=1}^{M}a_{ij}\left(\frac{D_{i}^{u}}{R_{ij}}+\frac{D_{i}^{u}C_{i}}{f_{ij}^{u}}\right)$$

Here, $f_{ij}^{u}$ represents the computational resources allocated to the *i*-th M-IoT device by the *j*-th UAV, which satisfies $\sum_{i=1}^{N}f_{ij}^{u}\leq F_{\max}^{u}$, where $F_{\max}^{u}$ denotes the maximum CPU computing frequency of each UAV.

The energy consumption generated by offloading the task of the *i*-th M-IoT device to the *j*-th UAV is defined as:

(11) $$E_{i}^{u}=\sum_{j=1}^{M}a_{ij}\frac{P_{ij}^{u}D_{i}^{u}}{R_{ij}}$$

#### 3.3.3. Cloud Computing Model

Assume that the communication link between the M-IoT device and the satellite has a fixed transmission rate $R_{s}$, which depends on the modulation and coding scheme and the bandwidth allocation of the satellite communication system. Let $f_{i}^{s}$ represent the cloud computing capability allocated to the *i*-th M-IoT device. Then, the transmission and processing delay for cloud computing can be expressed as:

(12) $$T_{i}^{s}=\frac{D_{i}^{s}}{R_{s}}+\frac{D_{i}^{s}C_{i}}{f_{i}^{s}}$$

Accordingly, the energy consumption for offloading the task of the *i*-th M-IoT device to the LEO satellite is:

(13) $$E_{i}^{s}=\frac{P_{i}^{s}D_{i}^{s}}{R_{s}}$$

Here, $P_{i}^{s}$ is defined as the transmission power for offloading the task of the *i*-th maritime IoT device to the LEO satellite.

In the partial offloading method, the total delay in the computing task is determined by the maximum values of the local execution and offloading execution delays. In addition, the total energy consumption for processing the task should include both local computation and the offloading energy. Therefore, the total delay and energy consumption for executing the task *D_i_* on the *i*-th M-IoT device are expressed as:

(14) $$T_{i}^{\mathrm{total}}=\max\left\{T_{i}^{l},T_{i}^{u},T_{i}^{s}\right\}$$

(15) $$E_{i}^{\mathrm{total}}=E_{i}^{l}+E_{i}^{u}+E_{i}^{s}$$

The weighted sum of the total delay and total energy consumption for the *i*-th M-IoT device at the *t*-th time slot represents the system cost for the *i*-th M-IoT device at time *t*, which can be defined as:

(16) $${\mathrm{Cost}}_{i}(t)=\omega_{T}T_{i}^{\mathrm{total}}+\omega_{E}E_{i}^{\mathrm{total}}$$

Here, $w_{T}$ and $w_{E}$ are weight parameters, representing the weight factors for the delay and energy consumption, respectively, satisfying the constraints $0⩽w_{T}⩽1$, $0⩽w_{E}⩽1$ and $w_{T}+w_{E}=1$.

### 3.4. Problem Description

In this section, to achieve the minimum system cost for all the M-IoT devices while satisfying the maximum available energy and tolerable delay constraints for the M-IoT devices, a related association control $a=\left\{a_{ij}\right\}$, task allocation $D=\left\{D_{i}^{l},D_{i}^{u},D_{i}^{s}\right\}$ transmission power control $P^{u}=\left\{P_{ij}^{u}\right\}$, and UAV computational resource allocation $f^{u}=\left\{f_{ij}^{u}\right\}$ optimization strategy for computational offloading is formulated.

(17) $$\begin{array}{ll} \min_{a,D,P^{u},f^{u}} & \sum_{t=1}^{T}\sum_{n=1}^{N}{\mathrm{Cos}t}_{i}(t)\\ s.t. & C1:T_{i}^{t}⩽T_{i},\forall i\in K\\ & C2:E_{i}⩽E_{i}^{\max},\forall i\in K\\ & C3:\sum_{j}a_{ij}f_{ij}^{u}⩽F_{j}^{u},\forall j\in N\\ & C4:D_{i}^{l}+D_{i}^{u}+D_{i}^{s}=D_{i},\forall i\in K\\ & C5:\sum_{j=1}^{N}a_{ij}⩽1,\forall i\in K\\ & C6:a_{ij}\in \{0,1\},\forall i\in K,j\in N\\ & C7:\sum_{j=1}^{N}a_{ij}P_{ij}^{u}⩽P_{i}^{\max},\forall i\in K \end{array}$$

Here, $E_{i}^{\max}$ is the maximum available energy for the *i*-th M-IoT device. The constraints in Equation (17) can be explained as follows: C1 indicates that each computational task should be completed before the tolerable delay; C2 ensures that the total energy consumption of each M-IoT device does not exceed its available energy; C3 limits the maximum CPU clock frequency for each M-IoT device at the UAV; C4 represents the constraint that each M-IoT device can only connect to one UAV; C5 is the association indicator variable for each M-IoT device; and C6 indicates the maximum transmission power for each M-IoT device.

This optimization problem is a non-convex mixed-integer optimization problem that cannot be solved by traditional methods, as it involves both continuous and discrete variables. Furthermore, the optimal offloading strategy depends not only on each device’s own strategy but also on the strategies of the other devices. The action space of the joint strategy increases exponentially with the number of devices and UAVs. Therefore, this section reformulates the problem as a POMDP and then designs a task offloading strategy based on the MADDPG to solve this optimization problem. Compared with DRL-based work, the MADDPG framework enables centralized training with decentralized execution, allowing agents to coordinate strategies despite the partial observational ability of the dynamic maritime environment. It can approach an optimized delay–energy trade-off [33].

## 4. MADDPG-Based Integrated Space–Air–Sea Network System Cost Minimization Algorithm

### 4.1. Partially Observable Markov Decision Process (POMDP) Strategy

In this section, the setups of the intelligent agents, state and action spaces, as well as the reward function, are introduced in detail. Each M-IoT device and each UAV is regarded as an intelligent agent, and the task offloading problem of each M-IoT device is modeled as a partially observable MDP (POMDP). In this process, the collaborative decision-making of the M-IoT devices and UAVs will impact the system cost. Therefore, the number of intelligent agents is set as *I*, I=M+N.

#### 4.1.1. State Space and Observation Space

At time *t*, the intelligent agents *i* observe the network state. The overall state space mainly consists of the positions of the IoT devices and UAVs, as well as information related to the devices’ tasks and transmission delays to the UAVs or LEO satellites, represented by $s(t)=\left\{p_{i}(t),q_{j}(t),R_{ij}(t)\right\}$. Considering that the system has partial observability, each intelligent agent can only observe part of the information in the overall state space. The observation space for an intelligent IoT device at time *t* is defined as $o_{i}(t)=\left\{p_{i}(t),R_{ij}(t)\right\}$, which indicates that the edge server can obtain position information through the GPS. The observation space for the UAVs is defined as $o_{i}(t)=\left\{q_{j}(t)\right\}$, meaning that the edge servers can obtain the position information.

#### 4.1.2. Action Space

To minimize the total delay and energy consumption of the M-IoT devices, the intelligent devices need to determine their task partitioning and corresponding transmission power $a_{i}(t)=\left\{a_{ij}(t),D_{i}^{l}(t),D_{i}^{u}(t),D_{i}^{s}(t)\right\}$, while the intelligent UAVs need to determine the association control and resource allocation strategy of the MEC server $a_{i}(t)=\left\{P_{ij}^{u},f_{ij}^{u}\right\}$. Therefore, the joint action space for the intelligent agents is $a(t)=\left\{a_{ij}(t),D_{i}^{l}(t),D_{i}^{u}(t),D_{i}^{s}(t),P_{ij}^{u},f_{ij}^{u}\right\}$.

#### 4.1.3. Reward Function

Generally, the reward in a real-time network is related to the objective function. In the optimization problem considered, the optimization goal is to minimize the total system cost, which is the weighted sum of the delay and energy consumption. To prevent the intelligent agents’ decisions from violating the computational resource limitations and UAV collision constraints, penalties are applied to agents that violate the constraints. Specifically, in multi-agent reinforcement learning, multiple agents collaborate to achieve the goal of minimizing the system cost. Therefore, all the agents share a common reward function, which is defined as

(18) $$r_{i}(t)=-{\mathrm{Cost}}_{i}(t)-\eta_{i}{\mathrm{Pen}}_{i}$$

Here, $\eta_{i}$ represents the binary penalty factor indicating whether an intelligent agent violates a constraint. If the constraint is violated, a penalty will be applied.

### 4.2. MADDPG Algorithm Framework

The MADDPG is a multi-agent reinforcement learning algorithm in which each intelligent agent collaborates with other agents through training two evaluation networks to achieve coordinated decision-making. Specifically, when an intelligent agent’s Actor network outputs an action, the states of the other agents will also be affected, impacting the next learning step’s strategy. Therefore, each agent’s decision is no longer independent. Additionally, by sharing a common experience replay buffer, each intelligent agent can fully consider the environment and the states of the other agents to make more rational decisions. This algorithm can solve the issue where traditional reinforcement learning algorithms struggle to converge in dynamic multi-agent environments.

**Figure 2:**
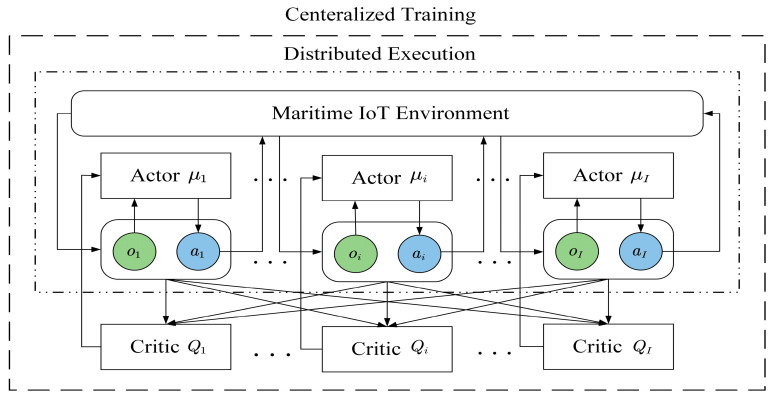
MADDPG framework description.

The framework of the MADDPG algorithm is shown in Figure 2, where each DDPG intelligent agent comprises Actor and Critic evaluation networks, along with the corresponding target networks. Additionally, all the agents share a common experience replay buffer, which stores the experiences encountered by each intelligent agent during training to update the network parameters. The target networks use a soft update method to update the parameters with a very small ratio to ensure the stability of the target networks, which accelerates convergence and enhances the algorithm’s stability.

The MADDPG algorithm adopts a combination of centralized training and decentralized execution. During the training phase, each intelligent agent sends its local observation $o_{i}(t)$ and action $a_{i}(t)$, and then all the agents’ observations and actions are combined into a state *s*(*t*) and sent to each agent. In this way, all the agents can exchange their local information simultaneously. Additionally, each agent’s Critic network is trained on the state and action of all the agents, which include the observations and actions of all the agents. During the testing phase, each agent only relies on its local observation $o_{i}(t)$ to execute the action that maximizes the cumulative rewards.

### 4.3. System Cost Minimization Algorithm for Space–Air–Sea Integrated Network Based on MADDPG

**Figure 3:**
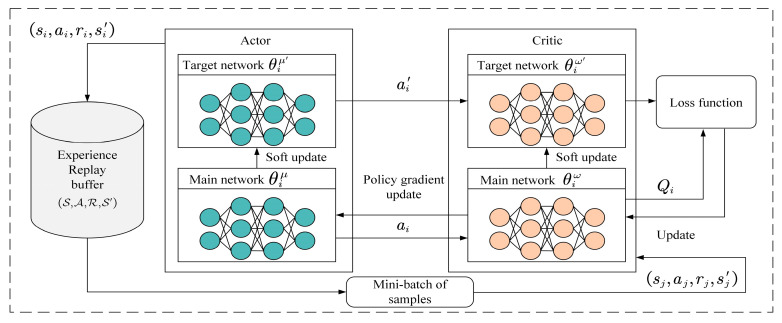
MADDPG algorithm flowchart.

The MADDPG algorithm proposed in this section comprises intelligent agents within the space–air–sea integrated network environment. The flowchart of the MADDPG algorithm is shown in Figure 3, with a detailed introduction to the training and testing process of intelligent agent *i*.

At each time step, each intelligent agent obtains its local observation $o_{i}(t)$ and executes its action $a_{i}(t)$, ultimately receiving its reward $r_{i}(t)$. Then, the environment updates the state and transitions to a new state $s_{i}(t+1)$. Each intelligent agent is equipped with an Actor network $\mu_{i}(o_{i}(t)|\theta_{i}^{\mu })$ and a Critic network $Q_{i}\left(s_{i}(t),a_{i}(t)|\theta_{i}^{\omega }\right)$, with the corresponding target networks $\mu_{i}^{\prime }\left(o_{i}(t)|\theta_{i}^{\mu^{\prime }}\right)$ and $Q_{i}^{\prime }\left(s_{i}(t),a_{i}(t)|\theta_{i}^{\omega^{\prime }}\right)$. The primary network and target network have the same structure but different parameters.

During the training phase, the Actor network of intelligent agent *i* takes the agent’s partial observation $o_{i}(t)$ as input, selects the action $a_{i}(t)$ based on the policy, and adds a certain amount of noise to the final output action, which can be defined as:

(19) $$a_{i}(t)=\mu_{i}\left(o_{i}(t)|\theta_{i}^{\mu }\right)+\Psi (t)$$

Here, Ψ(t) is the exploration noise that is used to encourage the agent to explore, and $\mu_{i}\left(o_{i}(t)|\theta_{i}^{\mu }\right)$ is the action output by the current Actor network of agent *n*.

In this way, the actions of all the agents can obtain rewards in the given environment, execute actions, and transition to the next state. Then, the combination of each agent’s state, action, reward, and next state (s(t),a(t),r(t),s(t+1)) is stored in the experience replay buffer D. When the accumulated count of experiences in the buffer reaches a certain amount, a mini-batch of samples is fed into the Actor and Critic networks of each agent. The sampled mini-batch is then input into the target Critic network to output the target network *Q* values as:

(20) $$y_{i}(j)=r_{i}(j)+\gamma Q_{i}^{\prime }\left(s(j+1),\mu_{i}^{\prime }\left(o_{i}(j+1)|\theta_{i}^{\mu^{\prime }}\right)|\theta_{i}^{\omega^{\prime }}\right)$$

Here, γ represents the discount factor, which is an important coefficient used to weigh current rewards against future rewards, and γ∈[0,1].

The Critic network is updated by minimizing the loss function, which is defined as:

(21) $$L\left(\theta_{i}^{\omega }\right)=\frac{1}{B_{b}}\sum_{j}{\left[Q_{i}\left(s(j),a(j)|\theta_{i}^{\omega }\right)-y_{i}(j)\right]}^{2}$$

Here, $B_{b}$ is the batch sample size.

For the Actor network, the network parameters are updated by maximizing the policy objective function, which is defined as:

(22) $$J\left(\theta_{i}^{\mu }\right)=\frac{1}{B_{b}}\sum_{j}Q_{i}\left(s(j),a(j)|\theta_{i}^{\omega }\right)$$

Finally, the parameters $\theta_{i}^{w^{\prime }}$ and $\theta_{i}^{\mu^{\prime }}$ of the target network are updated using a soft update method as follows:

(23) $$\theta_{i}^{\omega^{\prime }}\leftarrow \xi \theta_{i}^{\omega }+(1-\xi )\theta_{i}^{\omega^{\prime }}$$

(24) $$\theta_{i}^{\mu^{\prime }}\leftarrow \xi \theta_{i}^{\mu }+(1-\xi )\theta_{i}^{\mu^{\prime }}$$

Here, ξ is the soft update parameter, which is a smoothing factor used to control the update rate of the target network parameters, with ξ∈[0,1].

Algorithm 1 lists the steps for implementing the total cost minimization problem based on the MADDPG algorithm. As shown in Algorithm 1, the main idea of the proposed algorithm is to enable each M-IoT device and UAV to learn, via centralized training and decentralized execution, continuous offloading and resource allocation policies that minimize a weighted sum of the delay and energy consumption in a partially observable environment, which first initializes the parameters of each intelligent agent’s Actor network, Critic network, and two target networks. Then, the proposed algorithm undergoes E episodes, where each episode requires resetting the network environment to randomly generate the positions of the UAV and maritime IoT devices. Action exploration noise is added to ensure a normal distribution, which helps avoid suboptimal solutions due to local optimization during learning. At each time step, agents observe their state $o_{i}(t)$ based on the current strategy, select the optimal action $a_{i}(t)$ output by the Actor network, and obtain the *Q* value for that action from the Critic evaluation network and $Q^{\prime }$ from the Critic target network. Each agent *i* executes the action $a_{i}(t)$ and receives the corresponding reward $r_{i}(t)$ and next observation $o_{i}(t+1)$. Each agent then stores the experience tuple (s(t),a(t),r(t),s(t+1)) in the experience replay buffer. Next, a mini-batch of samples is randomly selected to update the Actor network parameters using the policy gradient and the Critic network using the minimum loss function. Finally, a soft update method is used to update the parameters of the target Actor and Critic networks. Then, considering the scalability of the proposed MADDPG-based algorithm, the CTDE architecture of the MADDPG enables scalable training by decoupling the critic’s polynomially growing joint state action evaluation from the decentralized, real-time execution using only local observations. The empirical results demonstrate near-linear cost scaling under fixed UAV resources, and practical measures such as parameter sharing, distributed replay buffers, federated updates and observation filtering can keep the training overhead and communication demands manageable while accommodating heterogeneous agent additions.
**Algorithm 1**: MADDPG-Based Maritime Computation Offloading and Resource Allocation Algorithm
1. Initialize the weight parameters of the Actor and Critic networks;
2. Initialize the weight parameters of the target Actor and target Critic networks;
3. **for** episode = 1 to E **do:**
4.  Reset the space–air–sea integrated network environment;
5.   Initialize the positions of the UAVs and maritime IoT devices;
6.   Each M-IoT device receives the initial state *O_i_*(*t*) and global state *s*(*t*) based on the task and network environment;
7.   **for** each time step *t* = 1 to T **do:**
8.     **for** *i* = 1 to N **do:**
9.        Each intelligent agent *i executes an* action based on the current policy;
10.       Executes the action *a_t_* = {*a*_1_(*t*), *a*_2_(*t*), … *a_n_*(*t*)} in the environment, receives the reward *r_t_* = {*r*_1_(*t*), *r*_2_(*t*), … *r_n_*(*t*)}, and transitions to the next state *s*(*t* + 1);
11.       **if** the experience replay buffer is not full **Then**
12.        Store (*s*(*t*), *a*(*t*), *r*(*t*), *s*(*t* + 1)) in the experience replay buffer D;
13.       **else**
14.        Replace the oldest tuple in D with the newly generated (*s*(*t*), *a*(*t*), *r*(*t*), *s*(*t* + 1));
15.        Randomly sample a mini-batch of *B_b_* samples from D;
16.        Minimize the loss function to update the Critic network parameters;
17.        Maximize the policy objective function to update the Actor network parameters;
18.        Update the target network parameters of the Actor and Critic networks;
19.      **end if**
20. $C_{A}=d_{o}H_{1}+H_{1}H_{2}+H_{2}d_{a}$      **end for**
21.   **end for**
22. **end for**

To evaluate the feasibility of the MADDPG-based algorithm in dynamic marine environments, this section analyzes the computational complexity of the proposed algorithm. The computational complexity of the algorithm is mainly composed of time complexity and space complexity.

First, the time complexity of the proposed algorithm while training is mainly composed of policy inference and parameter updating. The computational effort for policy inference can be approximated as follows:

$$C_{A}=d_{o}H_{1}+H_{1}H_{2}+H_{2}d_{a}$$

where *H*_1_ and *H*_2_ represents the size of the two hidden layers for a single Actor network, and $d_{o}$ and $d_{a}$ are the observation and action dimensions of the unit agent, respectively. Considering the input dimension of a single Critic network is $N(d_{o}+d_{a})$, where *N* denotes the number of agents, while two hidden layers are used, which is same as the Actor network, its combined forward and backward propagation overhead can be approximated as

$$C_{C}=N(d_{o}+d_{a})H_{1}+H_{1}H_{2}+H_{2}$$

Therefore, the policy inference of all the agents results in a computational effort of $O(NC_{A})$ in each time slot, and a single gradient update results in a computational effort of $O(NB(C_{A}+C_{C}))$, where *B* is the size of each sample batch in the experience replay buffer. Then, the training complexity of the proposed algorithm can be defined as follows:

$$O(ETN(C_{A}+B(C_{A}+C_{C})))$$

where *E* is the number of episodes, and *T* is the number of time slots. Then, in the online execution phase, the algorithm only needs to perform one forward propagation of the Actor network for each agent to generate the action; thus, the time complexity of the execution phase is $O(NC_{A})$, which omits batch sampling and all the backpropagation steps compared to the training phase.

Then, considering the space complexity of the proposed algorithm, the MADDPG requires the simultaneous maintenance of the experience playback buffers and four sets of neural network parameters. Considering that the buffer capacity is R and each sample contains forward and backward observation vectors, action vectors, and a scalar reward, then the overall buffer storage overhead can be approximated as follows:

$$O(R(2Nd_{o}+Nd_{a}+1))$$

While considering the network parameters, the corresponding number of parameters for each Actor and Critic network is *C_A_* and *C_C_*. The total of four networks with the total storage requirements can be defined as follows:

$$O(4N(C_{A}+C_{C}))$$

Therefore, the overall space complexity of the proposed algorithm can be summarized as

$$O(RN(d_{o}+d_{a}))+O(N(C_{A}+C_{C}))$$

## 5. Algorithm Performance Evaluation and Comparison

This section verifies the performance of the proposed algorithm through simulation experiments. First, it sets up the simulation parameters, then it describes the comparison with other algorithms, and finally, it verifies the performance of the proposed MADDPG algorithm through simulation experiments and compares it with baseline algorithms.

### 5.1. Simulation Setup

The simulation experiment in this section uses the PyCharm 2024.3.1 and PyTorch 2.7.1 frameworks to implement the proposed MADDPG algorithm. It primarily studies a cloud–edge collaborative system composed of one LEO satellite, multiple UAVs, multiple MEC servers, and multiple M-IoT devices. It is assumed that each UAV is equipped with an MEC server, and the M-IoT devices can offload tasks to the MEC servers via wireless channels for computation. The maritime domain is assumed to be 500 × 500 m^2^, and the positions of the maritime IoT devices are randomly distributed within this domain. The input data size of the computation tasks is uniformly distributed between [1, 5] Mbits, with the required CPU cycles ranging from [10^8^, 10^9^]. The specific simulation parameters are shown in Table 1.

In the MADDPG algorithm, the Actor network and Critic network are neural networks with two hidden layers, each containing 400 and 300 neurons. The discount factor *γ* is set to 0.99, the experience replay buffer D size is 1000, the minimum batch size $B_{b}$ is 100, and the soft update rate ξ is 0.001. Additionally, the learning rates for updating the Critic and Actor networks for all the intelligent agents using the ADAM optimizer are 0.001 and 0.002, respectively. To evaluate the performance of the proposed algorithm, this section introduces four baseline algorithms for comparison:

1. All-local algorithm: Each M-IoT device executes computational tasks independently with its maximum computing capability, without offloading any tasks to the UAV or LEO satellite.
2. All-offload algorithm: Each M-IoT device completely offloads its task data to the UAV, where the MEC server on the UAV performs assisted computing.
3. Random algorithm: Each M-IoT device randomly allocates computational tasks between local execution and execution on the UAV.
4. D3QN algorithm: This algorithm uses the D3QN algorithm to optimize the MEC server resource allocation and offloading strategies. Specifically, a single D3QN network is used to decide the offloading strategy for intelligent agents, with the action space, state space, and reward space combining the actions, states, and rewards of all the maritime IoT devices and UAVs.

### 5.2. Simulation Result Analysis

**Figure 4:**
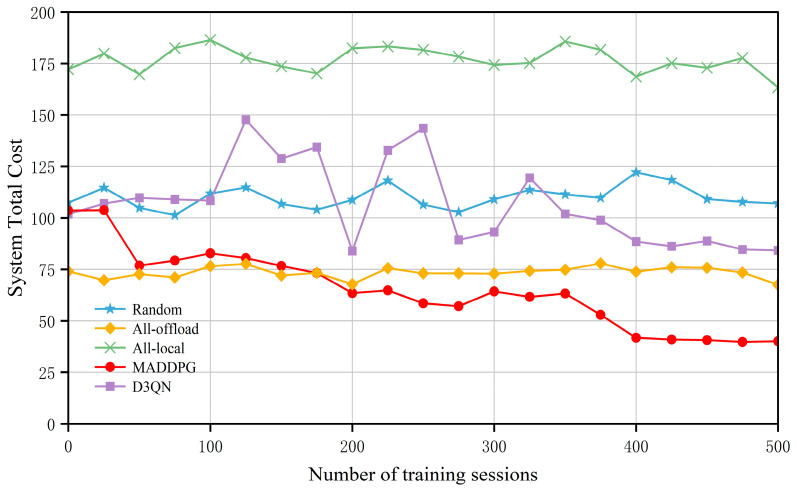
Comparison of convergence across different algorithms.

Firstly, to verify the convergence performance of the MADDPG and other baseline algorithms in this scenario, a total of 500 training episodes were set. Figure 4 shows the convergence of the total system cost under the different algorithms, with the horizontal axis representing the number of training episodes and the vertical axis representing the total system cost. The All-local and All-offload algorithms remain relatively stable, while the system cost achieved by the Random algorithm is unstable due to its randomness and requires a long period of training to yield convergence. The D3QN algorithm shows relatively large fluctuations and takes a longer training time to achieve convergence.

Compared to these baseline algorithms, the MADDPG algorithm performs the best. Specifically, in the early stages of iteration, the MADDPG algorithm experiences significant fluctuations. This is mainly because, during the initial learning phase, the neural network is in the exploration stage, with the agents yet to collect sufficient experience samples from the replay buffer, leading to considerable instability. As the number of training iterations increases, the agents continuously interact with the environment, exploring the impact of offloading locations on the reward function. They consistently search for the optimal offloading strategy and learn effective computation offloading and resource allocation policies, achieving the goal of reducing the total system cost. After 400 training episodes, the MADDPG algorithm tends to stabilize, primarily due to the centralized training and distributed execution strategy adopted by MADDPG. All the agents can share each other’s state observation information, enabling them to make more informed decisions. Compared to traditional reinforcement learning methods, this technique is more suitable for dynamic and complex environments, allowing for flexible offloading and resource allocation decisions based on user mobility, thus reducing the total system cost.

**Figure 5:**
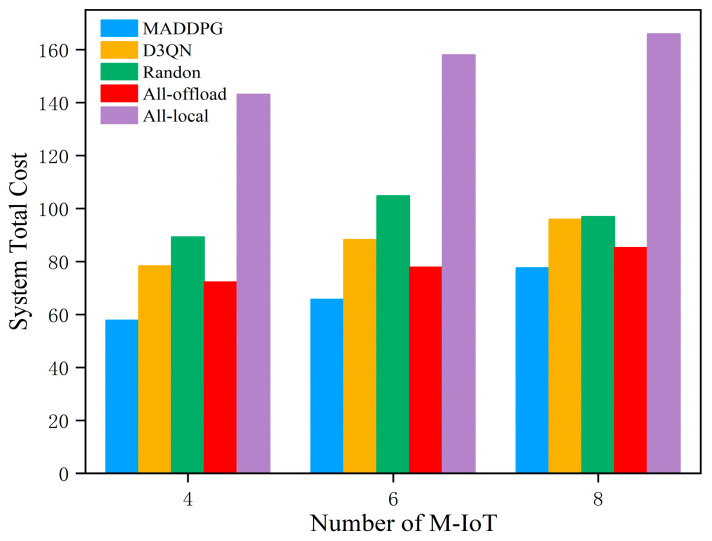
Comparison of total system costs with different numbers of M-IoT devices.

Secondly, with the other parameters kept constant, the variation in the total system cost under different numbers of M-IoT devices was verified, as shown in Figure 5. The horizontal axis represents the number of M-IoT devices, while the vertical axis represents the total system cost. In this experiment, the M-IoT device quantities were set to 4, 6, and 8. As the number of devices increases, the total system cost also increases. Figure 5 shows that the All-local algorithm incurs the highest system cost. This is because the computational capacity of the local devices is limited, leading to high system costs due to restricted device computing resources. Due to the randomness in selection, the Random algorithm yields inconsistent strategies, resulting in fluctuating system costs that lack stability.

**Figure 6:**
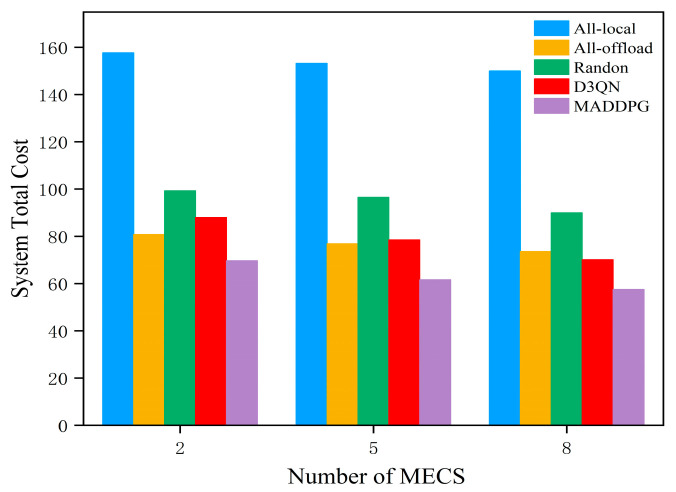
Comparison of total system costs with different numbers of MEC servers.

Figure 6 illustrates the impact of different numbers of MEC servers on the total system cost, with the horizontal axis representing the number of MEC servers and the vertical axis representing the total system cost. From Figure 6, it can be observed that as the number of MEC servers increases, the overall system cost gradually decreases. This is mainly because, with an increased number of MEC servers, IoT devices can offload more tasks to MEC servers simultaneously, significantly enhancing the system performance. From an algorithmic perspective, the total cost generated by the local offloading strategy is the highest. The random offloading strategy results in a large variation in the system costs due to the different choices made by the strategy. The D3QN algorithm, which uses neural networks for training, has achieved a relatively optimal model; however, since it operates with only a single agent, it can easily fall into a local optimum, failing to find a global optimal solution. In contrast, the MADDPG algorithm performs better than the D3QN algorithm, yielding an optimized offloading strategy that demonstrates superior optimization results and plays a significant role in resource allocation and load balancing for the servers. Therefore, setting an appropriate number of MEC servers suitable for the current network conditions is crucial, as it can greatly improve the system performance.

**Figure 7:**
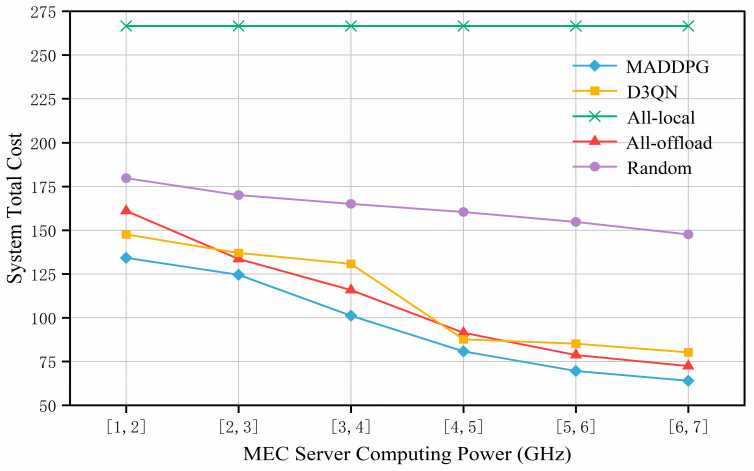
Comparison of total system costs with different MEC server computing capacities.

Figure 7 shows the changes in the total system cost under different computing capacities of MEC servers. As the computing capacity of the MEC servers increases, the total system cost shows a downward trend for all the algorithms except the All-local algorithm. This is mainly because the UAV can allocate more computing resources to the maritime IoT devices, thereby reducing the devices’ computational energy consumption and latency, achieving the goal of lowering the total system cost. The total system cost of the All-local algorithm remains constant because the delay and energy consumption of the devices executing tasks locally are unrelated to the increase in the computing capacity of the MEC servers.

For the MADDPG algorithm, the system cost continues to decrease and is consistently lower than the system costs of the All-local, All-offload, Random, and D3QN algorithms. However, when the MEC computing capacity increases to a certain level, limited communication resources constrain the reduction in the total system cost, and the downward trend of the MADDPG algorithm gradually slows down. In summary, the MADDPG algorithm proposed in this section effectively allocates computing resources and learns better offloading strategies through cooperative learning among multiple agents, thereby reducing the total system cost and achieving better system performance.

Finally, the contributions of this paper are summarized in Table 2.

## 6. Conclusions

Considering the limited energy and resources of the nodes in the system, this section proposes a collaborative cloud–edge joint task offloading and resource allocation optimization problem, utilizing UAVs and LEO satellites to provide computational offloading services for maritime IoT devices, with the aim of minimizing the total system cost. Since this optimization problem is a mixed-integer nonlinear programming problem, this section introduces an MADDPG algorithm based on multi-agent reinforcement learning, which can better find the optimal strategy. Simulation experiments verify the convergence of the proposed algorithm. Compared with other baseline algorithms, the MADDPG algorithm proposed in this section achieves a lower overall system cost under the same conditions. Meanwhile, the limitations are considered. The current model assumes ideal channel state information and static energy constraints. We will consider the effects of channel estimation errors and battery degradation in future work.

## Figures and Tables

**Table 1:** Simulation parameter setup.

Parameter	Value
Number of M-IoT devices	3
Number of UAVs and LEO satellites	5, 1
Bandwidth *B_ij_*	1 MHz
Noise power *N*_0_	−100 dBm
$\mathrm{Transmission}\;\mathrm{power}\;\mathrm{of}\;M-\mathrm{IoT}\;\mathrm{devices}\;p_{ij}^{u}$	[1, 3] W
$\mathrm{Maximum}\;\mathrm{transmission}\;\mathrm{power}\;P_{i}^{\max}$	3 W
$\mathrm{Task}\;\mathrm{data}\;\mathrm{size}\;D_{i}$	[1, 5] MB
$\mathrm{Maximum}\;\mathrm{tolerable}\;\mathrm{delay}\;T_{i}$	0.5 s
$\mathrm{Local}\;\mathrm{computing}\;\mathrm{capability}\;f_{i}^{l}$	1 GHz
$\mathrm{Edge}\;\mathrm{server}\;\mathrm{computing}\;\mathrm{capability}\;f_{ij}^{u}$	[1, 5] GHz
$\mathrm{Cloud}\;\mathrm{server}\;\mathrm{computing}\;\mathrm{capability}\;f_{i}^{s}$	15 GHz
$\mathrm{Energy}\;\mathrm{efficiency}\;\mathrm{coefficient}\;\kappa_{i}$	10^−27^
Weight factors *ω_T_*, *ω_E_*	0.5, 0.5
UAV flight altitude *H*	100 m

**Table 2:** Summary of the contributions.

Refs	Algorithm	Optimization Problem	Objective	Performance Metrics
[16]	Lyapunov-based stochastic optimization and delayed online learning	Online task-routing/offloading	Minimize long-term expected task-completion delay	Average delay, satellite queue length
[17]	Gradient-free online scheduling	Fog-CPU allocation for marine-sensor data compression	Maximize comm.-resource efficiency under QoS	Bandwidth saving ratio, data-quality (PSNR) satisfaction
[18]	Hopcroft–Karp matching and real-time heuristics	Voyage-based offloading via shipborne edge nodes	Jointly minimize delay, energy and traffic cost	Task delay, UE energy, backhaul cost
[19]	Improved Hungarian algorithm	Multivessel offloading decision and server selection	Min-weighted sum (Energy + Delay)	Total terminal energy, execution latency
[20]	Multi-agent Deep-Q-Learning	Dynamic power control in dense wireless links	Maximize weighted sum-rate utility	Network sum-rate, spectral-efficiency gain
[21]	REINFORCE/DQL/DDPG (actor–critic)	Downlink power allocation for multi-cell IBC	Maximize instantaneous and average sum-rate	um-rate, robustness to CSI error
[22]	Double Deep-Q-Learning with outage guard	Power allocation in 6G interference channel	Maximize sum-rate under link-outage constraints	Sum-rate, outage probability
[23]	Federated multi-agent DRL	Joint AeBS deployment and offloading	Minimize task delay and energy for aerial MEC	Average delay, node energy, convergence speed
This work	MADDPG (continuous actor–critic, centralized training/decentralized execution)	Joint offloading ratio, UAV–terminal association, Tx-power, CPU-frequency allocation in Space-Air-Sea MEC networks	Minimize weighted system cost (Energy + Delay)	Energy–delay cost, convergence rate, overall efficiency

## Data Availability

The original contributions presented in this study are included in the article. Further inquiries can be directed to the corresponding author.
