# Correction: Gao, H.; Yu, Q. Research on Computation Offloading and Resource Allocation Strategy Based on MADDPG for Integrated Space–Air–Marine Network. *Entropy* 2025, *27*, 803

**DOI:** 10.3390/e27121244

**Published:** 2025-12-09

**Authors:** Haixiang Gao, Qianlian Yu

**Affiliations:** College of Information Engineering, Shanghai Maritime University, Shanghai 200135, China; 17317318573@163.com

**Addition of an Author**

Qianlian Yu was not included as an author in the original publication [1]. Authors contributed equally to the original paper should be included. The corrected Author Contributions statement appears here. The authors state that the scientific conclusions are unaffected. This correction was approved by the Academic Editor. The original publication has also been updated.

Conceptualization, H.G. and Q.Y.; methodology, Q.Y.; software, H.G.; validation, H.G. and Q.Y.; formal analysis, Q.Y.; investigation, H.G.; resources, H.G. and Q.Y.; data curation, Q.Y.; writing—original draft preparation, H.G. and Q.Y.; writing—review and editing, H.G.; visualization, H.G.; supervision, H.G. and Q.Y.; project administration, H.G. and Q.Y.; funding acquisition, H.G. and Q.Y. All authors have read and agreed to the published version of the manuscript.
